# Parameter Estimation of Two Spiking Neuron Models With Meta-Heuristic Optimization Algorithms

**DOI:** 10.3389/fninf.2022.771730

**Published:** 2022-02-16

**Authors:** Amr M. AbdelAty, Mohammed E. Fouda, Ahmed Eltawil

**Affiliations:** ^1^Engineering Mathematics and Physics Department, Faculty of Engineering, Fayoum University, Faiyum, Egypt; ^2^Computer, Electrical and Mathematical Sciences and Engineering Division, King Abdullah University of Science and Technology (KAUST), Thuwal, Saudi Arabia; ^3^Center for Embedded & Cyber-Physical Systems, University of California, Irvine, Irvine, CA, United States; ^4^Nanoelectronics Integrated Systems Center (NISC), Nile University, Giza, Egypt

**Keywords:** spiking neuron model, meta-heuristic optimization algorithms, leaky integrate and fire (LIF), adaptive exponential (AdEx) integrate and fire, *in-vitro* data, cuckoo search optimizer, marine predator algorithm

## Abstract

The automatic fitting of spiking neuron models to experimental data is a challenging problem. The integrate and fire model and Hodgkin–Huxley (HH) models represent the two complexity extremes of spiking neural models. Between these two extremes lies two and three differential-equation-based models. In this work, we investigate the problem of parameter estimation of two simple neuron models with a sharp reset in order to fit the spike timing of electro-physiological recordings based on two problem formulations. Five optimization algorithms are investigated; three of them have not been used to tackle this problem before. The new algorithms show improved fitting when compared with the old ones in both problems under investigation. The improvement in fitness function is between 5 and 8%, which is achieved by using the new algorithms while also being more consistent between independent trials. Furthermore, a new problem formulation is investigated that uses a lower number of search space variables when compared to the ones reported in related literature.

## 1. Introduction

The tuning of spiking neuron model parameters is a challenge that started with a manual trial-and-error approach and evolved into an automatic approach as the availability of computational resources increased. An optimization problem that aims at tuning the parameters of a spiking neuron model is composed of two independently chosen components: the fitness function and the search algorithm. The fitness function is a quantitative measure of how well the model response fits the measured data. The optimization/search algorithm job is to explore the search space to find the optimal parameters within a short amount of time. The choice of these two components is independent except for the case of the multi-objective optimization approach (Van Geit et al., 2008).

The leaky-integrate and fire (LIF) model is widely used for studying neural systems. In this model, the membrane potential of a neuron is described as a function of the synaptic input currents. A spike is generated when the membrane potential crosses a threshold. However, in the original model, the physical changes in the conductances are not part of the model. The LIF model dates back to 1907 when Lapicque suggested a circuit model of the neuron membrane voltage that is composed of a resistor and a parallel capacitor (Lapique, 1907). The capacitor represents the integrator, and the resistor is the leaky element of the model. The membrane potential is reset for a refractory period after producing a spike. This simplified model enabled Lapicque to derive the firing rate as a function of the stimulating voltage magnitude. This model separated two-time scales: the slow sub-threshold integration and the fast spike generation (Burkitt, 2006). The most biologically plausible spiking neuron model is the Hodgkin-Huxley one which is an example of multi-compartmental, conductance-based models (Hodgkin and Huxley, 1952; Izhikevich, 2004). Their work led to an immediate interest in electro-physiology, for which they shared the 1963 Nobel prize in Physiology or Medicine (Schwiening, 2012). In between these two extremes lie many models that are evolved from IF models to improve their fitting capabilities. One of the major additions to these models is the adaptation variables. Examples of these models are, in order of complexity, the IF model with an adaptive current, the adaptive threshold IF model (Gerstner et al., 2014), and the adaptive quadratic (Izhikevich) IF models (Izhikevich, 2003). Many of these models were investigated in the problem of automatic fitting to experimental spiking neuron recordings. Simple models are more capable of accurate fitting to spike timing. In contrast, complex models, like the HH model, are more challenging to optimize due to their numerous parameters and simulation time (Rossant et al., 2010; Lynch and Houghton, 2015). All these efforts take part in solving the crucial neuro-science quest to figure out the input-output function of the neuron. Examples of recent effort in this direction can be found at Ujfalussy et al. (2018), Beniaguev et al. (2021), Harkin et al. (2021).

The optimization approach for spiking neuron models depends on the model itself and the nature of the experimental data. In some cases, the injected current can be changed freely, and this is used to isolate some model parameters via an analytical method. However, this is not always the case, and a more generic approach is needed for this problem. Thus, researchers tend to use global heuristic optimizers to search the parameter space without the need to calculate the gradient of the performance metric (Rossant et al., 2010; Lynch and Houghton, 2015). This property is important in case of fitting problems that are based on spike trains where the metrics have discontinuous nature. Examples of spike train metrics are the van Rossum metric, the inter-spike-interval distance, and the Γ coincidence factor (Kreuz et al., 2020). A genetic algorithm and particle swarm optimization are the main optimization algorithms used to identify the parameters of spiking neural models in the literature (Rossant et al., 2010; Lynch and Houghton, 2015). Recently, several other nature-inspired optimization algorithms have been presented and proven to give better results in many applications (Yousri et al., 2018, 2021). Examples of these algorithm are the cuckoo search optimizer (Gandomi et al., 2013), the marine predator algorithm (Faramarzi et al., 2020), and the gray wolf optimizer (Yousri et al., 2020) (check Abd Elaziz et al., 2021 for a recent review of these algorithms). However, these newly introduced algorithms have not been used to tackle the problem of the estimation model parameter of spiking neurons. In this paper, the genetic algorithm (GA), marine predator algorithm (MPA), cuckoo search optimizer (CS), fractional-order cuckoo search optimizer (FOCS), and particle swarm optimizer (PSO) are used to fit the spike timing of the *in vitro* current injection responses provided within the Quantitative Single Neuron Modeling Competition (QSNMC) dataset (Naud et al., 2009). Two different optimization approaches are discussed for each of the two spiking neuron models under investigation.

The main contributions of this paper can be summarized as follows:

- Applying new meta-heuristic optimization algorithms, other than the ones commonly used in literature, to tackle the problem of fitting spike times of experimental neuron recordings. This results in more accurate fitting and validation coincidence factors.
- Introducing a new problem formulation that achieved a higher mean coincidence factor than the traditional approach.
- Providing a statistical analysis (mean, standard deviation, and coefficient of variation) on the coincidence factor and model several parameters for two different spiking neuron models.

This paper is organized as follows: Section 2 discusses the QSNM2009 dataset and related works. Then, Section 3 discusses the spiking neuron models under investigation. Section 4 formulates two optimization problems and reviews the five meta-heuristic optimization algorithms whose results are compared in this work. The results and their discussion are presented in Section 5, while the concluding remarks and suggested future research directions are in Section 5.

## 2. QSNMC2009 Dataset

The aim of the QSNMC, in its 2009 version, was to offer a framework for comparing fitting algorithms and neuron models by providing a common set of neuronal recordings for fair comparison (Naud et al., 2009). It had four challenges: A, B, C, and D, and *Challenge A* is the focus of this manuscript. This challenge was predicting the spike timing of regular (not fast) spiking of the L5 pyramidal cell as it responds to a stimulation current that is synthesized in a way to make it similar to the current observed in *in-vivo* conditions. The current stimulus had two main parts. The first one is 17.5 s long and consisted of four-step currents of a 2 s duration each, and between steps, there were 2 s of rest. The first current step is hyper-polarizing (negative current), and the other three are depolarizing (positive current) with increasing intensity. Following the current steps, a white noise injection was made for 2 s.

A simulated excitatory and inhibitory spike train was then injected for a duration of 42.5 s. Six spike trains were used to compile this stimulant. The first three were convolved with an exponential decay with a 2 ms time constant, while the other three were also convolved with an exponential decay but with a time constant of 10 ms. These six time series were merged by a weighted sum whose weights were chosen so as to stimulate the neuron to spike at a frequency between 5 and 10 Hz, which resembles *in-vivo* conditions.

The training set was chosen to be the first 38 s, and the test set was the last 22 s. The injected current, with a 60 s length, was provided to the participants. However, the voltage traces of the 13 recordings were provided for only the first 38 s (the training duration).

### 2.1. Related Works

There are a few papers that used the experimental spiking neuron data provided by the International Neuroinformatics Coordinating Facility (INCF) at their QSNMC in 2009. The details and results of this competition are outlined in Gerstner and Naud (2009) and Naud et al. (2009, 2011, 2012). The dataset was made available at the Github repository of the INCF to allow further research on the problem (Gerkin, 2009). Although many papers cite this dataset, only a few papers used it to identify the parameters of spiking neuron models (Rossant et al., 2010, 2011; Russell et al., 2010; Yamauchi et al., 2011; Mitra et al., 2013; Lynch and Houghton, 2015).

One of the earliest papers to use the QSNMC2009 dataset is Rossant et al. (2010). The authors used a particle swarm optimizer (PSO) as the global optimization algorithm and the coincidence factor (Γ), defined in Section 3.2, as the objective function. They also provided a model fitting library integrated with the Brian neuron simulator and capable of running in parallel on GPUs. They proposed an online approach to calculate the Γ factor where the spike coincidences are counted as the model is simulated, and not post-simulation as usual. The model was simulated using time slicing, with an overlapping concept to parallelize the evaluation of the model further. The optimization procedure was tested on the synthetic data of the LIF model with an adaptive threshold by injecting an Ornstein-Uhlenbeck process current for 500 ms. The coincidence window was set to δ = 0.1 ms for this test. A perfect match was obtained within a few iterations. The parameter values were within ±15% of the ones used for synthesis and were found to be within ±3% when the number of particles was increased. However, the number of particles was not reported. For the experimental data, results were summarized for the dataset of Challenges A and B of QSNMC2009. *Challenge A* is for regular spiking neurons, and *Challenge B* is for fast-spiking neurons. The data of each recording in each challenge were divided into training and testing periods, each of which had a duration of 10 s. For *Challenge A*, the optimization was performed over the period from 17.5 s to 28 s, and the coincidence factor was reported for 28 s to 38 s with a coincidence window of δ = 4 ms. The intrinsic reliability (Γ_*in*_), defined in Section 3.2, was explicitly reported in this paper to be Γ_*in*_ = 0.78 and Γ_*in*_ = 0.74 for Challenges A and B, respectively, but neither the source for these values nor the period over which these values were computed was provided. A time shift parameter was used to shift the model spike to align with the recorded spike. This happens because the spike times were recorded as the times when the membrane voltage crossed zero. The optimization was performed on each record independently, and the mean and standard deviation of the coincidence factor were reported. For example, the adaptive exponential IF model achieved Γ = 0.51 ± 0.04(65%) for *Challenge A* and Γ = 0.76 ± 0.05(102%) for *Challenge B*. The values in brackets are the normalized value with respect to the intrinsic reliability.

In the review (Rossant et al., 2011), the authors applied their previously developed toolbox, which was based on Brian and used efficient parallelization concepts, to the QSNMC2009 dataset in order to estimate many of the parameters of the models that participated in the competition. The authors reported that their results were different from the ones reported in the competition due to the fact that they used only the available dataset and divided it into fitting and testing parts, whereas the entire available data was used for fitting in the competition. However, the authors did not specify the time periods used for fitting and testing. They developed and used a parallel version the Covariance Matrix Adaptation Evolution Strategy as the optimization algorithm suitable for GPU and multiple CPU simulations, and the coincidence factor as the objective function.

An optimization method based on the maximum likelihood (ML) function was proposed for the Mihalas–Niebur spiking neuron model in Russell et al. (2010). To validate their method, the authors simulated 250 ms of tonic bursting and used it as the target spike pattern. The results of optimization of the synthetic case were described qualitatively to be an almost perfect match for spike timing. One second of the QSNMC2009 dataset was used to configure/predict the parameters of the model using the ML function. First, the spike time of each repetition of the 13 recordings was extracted as the time when the voltage trace crossed 0 mV. The optimization was then performed on the 13 recordings. However, the authors have not reported either the optimization algorithm or the period they have optimized over. They reported that their resulting voltage response was a 1.2 ms average difference in its inter-spike intervals compared with the experimental response, with a standard deviation of 1.12 ms. As the recorded data had an average inter-spike interval of 39.4 ms, this error is approximately 3%.

An augmented Multi-timescale Adaptive Threshold (MAT) model was proposed in Yamauchi et al. (2011) by adding voltage dependency to the adaptive threshold in order to increase the variety of its firing patterns. The original MAT model won first place in QSNMC2009 competition, *Challenge A*. The authors did not use synthetic data to validate their method. The AugMAT model parameters were adjusted to match the data of 10 out of 13 voltage responses of the QSNMC2009, and then validated using the rest of the trials. The 10 trials were randomly selected, but the details of the parameter tuning were omitted, and the time window of evaluating Γ was not mentioned. The paper is more focused on introducing the AugMAT model and its new spiking patterns than the parameter identification problem. This process was repeated 100 times, and the coincidence factor, Γ, was used to assess the performance of the model using a coincidence window of 4 ms. The AugMAT model achieved Γ = 0.84, while the original MAT model achieved Γ = 0.77 in their predictive performance. Augmented MAT was superior at different coincidence windows (2 ms to 10 ms).

In Mitra et al. (2013), the authors used a gradient descent algorithm to estimate the parameters of the AugMAT model using a synthetic dataset. The stimulating current had a length of 1 s and was generated by a sum of different exponentials to emulate the stimulus received from a dendrite tree. The search space in the synthetic case was five-dimensional, and the gradient descent reached the minimum within a finite number of steps. The authors proposed a differentiable performance function that can be viewed as a special case of the Van-Rossum metric. The performance function is given as


(1) $$\begin{array}{l} \zeta =\frac{1}{t_{f}^{2}}\int_{0}^{t_{f}}{\left[\psi_{1}\left(t\right)-\psi_{2}\left(t\right)\right]}^{2}dt, \end{array}$$

where ψ_*i*_(*t*) is the convolution of the spike train of the neuron number *i* with the Heaviside unit step function. A hybrid technique was proposed for the parameter estimation of the AugMAT model applied to the experimental spike train dataset provided with QSNMC2009. They chose to use the same part of the dataset discussed in Yamauchi et al. (2011), which is a 4s window from the time instance 17.5 s to 21.5 s. However, the record number, out of 13, was not specified. The hybrid technique used a gradient descent and a Nelder–Mead algorithm. The gradient descent started at a randomly chosen initial point using the ζ performance index until a previously specified number of iterations was reached, and the Nelder–Mead then used the result of the previous phase as the initial point of its search using the coincidence factor (Γ). This hybridization was aimed to overcome the limitations of using each of these algorithms alone. The simulation was conducted 100 times with different random initial parameters for the hybrid method (GD+NM) and the NM alone. The statistics of the results are 0.65 ± 0.09 and 0.55 ± 0.1 for GD+NM and NM, respectively.

The latest paper to use the QSNMC2009 dataset is Lynch and Houghton (2015). The authors used the genetic algorithm for optimization and the Van-Rossum metric as the objective function. The validity of the optimization procedure was tested on the adaptive exponential integrate and fire model (aEIF) by using 4 s of synthetic data generated from a random input current signal. The first 2 s were for training, and the last 2 s were for validation. This test was run 20 times. The population size of the genetic algorithm was set to 240, and the number of iterations was set to 1, 000. Three synthetic tests with the time constant, τ, of the Van-Rossum metric were made. The first test used varying values of τ that start with half of the simulation time and gradually decrease until it reaches the mean inter-spike interval. In the second one, τ was set to be the simulation time; in the third one, τ was set to be the mean inter-spike interval. When comparing the results based on the coincidence factor, the larger time scale had the lowest Γ, the short time scale was better, and the varying time scale was the best. It is worth mentioning that the mean Γ factor value for a varying time scale case was not equal to 1. For the experimental data, the authors used the same 20.5 s used in Rossant et al. (2010), starting at 17.5 s, where the first 10.5 s were used for fitting, and the last 10 s were used for validation. The spike time was determined to be the times in the trace where the voltage crosses a certain value, but this threshold value was not specified in the paper. The genetic algorithm was set to run for 800 iterations in the experimental data case for 10 interdependent runs. The authors tested five neuron models in that paper: the aIF, atIF, aEIF, a2EIF, and Izhikevich models. The aEIF and a2EIF models achieved the top Γ factor values.

## 3. Neuron Models and Metrics

### 3.1. Spiking Neuron Models

In this work, we consider two different neuron models: the adaptive exponential integrate and fire (aEIF) model (Brette and Gerstner, 2005), and the adaptive-threshold with adaptation variable integrate and fire (aTIF-W) model (Koch, 2004). The latter is a combination of the adaptive threshold leaky integrate and fire model and the leaky integrate and fire model with an adaptation variable. These two models represent different variations of the integrate and fire model with almost the same number of parameters. The aEIF model is written as


(2a) $$\begin{array}{l} \tau_{m}\frac{dv}{dt}=\left(E_{L}-v\right)+\Delta_{T}e^{\left(v-v_{T}\right)/\Delta_{T}}-w+RI \end{array}$$

(2b) $$\begin{array}{l} \tau_{w}\frac{dw}{dt}=b\left(v-E_{L}\right)-w, \end{array}$$

where *v* is the membrane potential, *I* is the input current, *R* is the leak resistance, τ_*m*_ is the membrane time constant, *E*_*L*_ is the resting potential, *V*_*T*_ is the spike threshold, Δ_*T*_ is the slope factor, *w* is the adaptation voltage, τ_*w*_ is the adaptation time constant, and *b* is the sub-threshold adaptation. The sharp reset condition is given at a constant cut-off voltage *v*_*c*_ as follows: when *v*(*t*) > *v*_*c*_, then


(3) $$\begin{array}{l} v\to v_{r},w\to w+\alpha , \end{array}$$

where *v*_*r*_ is the reset voltage, and α is the spike-triggered adaptation. It is worth mentioning that, when *v*(*t*) ≈ *E*_*L*_ and *I* = 0, the adaptation voltage, *w*, decays to zero. Therefore, the point (*E*_*L*_, 0) can be considered an equilibrium point of this system of equations by taking into consideration that the exponential term is very small in this case.

The adaptive threshold IF model with adaptation current is given as


(4a) $$\begin{array}{l} \tau_{m}\frac{dv}{dt}=\left(E_{L}-v\right)-w+RI, \end{array}$$

(4b) $$\begin{array}{l} \tau_{w}\frac{dw}{dt}=b\left(v-E_{L}\right)-w, \end{array}$$

(4c) $$\begin{array}{l} \tau_{t}\frac{dv_{c}}{dt}=c\left(v-E_{L}\right)-v_{c}. \end{array}$$

where *v*_*c*_ is the cut-off voltage, *c* is the sub-threshold adaptation factor of the cut-off voltage, and τ_*t*_ is the cut-off voltage time constant. The sharp reset condition is given at a time-dependent cut-off voltage *v*_*c*_(*t*) as follows: when *v*(*t*) > *v*_*c*_(*t*), then


(5) $$\begin{array}{l} v\to v_{r},w\to w+\alpha ,v_{c}\to v_{c}+\beta , \end{array}$$

where α and β are the spike-triggered adaptation of the adaptation voltage and cut-off voltage, respectively. Similar to the aEIF model, the point (*E*_*L*_, 0, 0) is an equilibrium point of the system.

In this work, the spike time is recorded at the instant when the sharp reset condition is applied. It is important to note that the chosen models are not the best performing models in the literature. However, their simplicity and popularity make them good candidates when the goal is to compare optimization algorithms, which is one of the objectives of this work.

### 3.2. Identification Metric

The performance was evaluated based on the coincidence factor Γ. Several quantities are needed to calculate Γ, including *N*_*data*_ and *N*_*model*_, which are the number of spikes in the target and model responses, respectively. *N*_*coinc*_ is the number of coincident spikes between the model response and the target. A target spike is considered coincident if there is a least one spike in the model that matches the target spike time within the coincident window (δ = 4*ms* in the case of QSNMC2009). The Γ factor between two spike trains is given as (Jolivet et al., 2008; Naud et al., 2009)


(6) $$\begin{array}{l} \Gamma =\frac{1}{1-2\delta f}\frac{N_{coinc}-2\delta N_{data}f}{N_{data}+N_{model}}, \end{array}$$

where *f* is the average firing rate of the target spike train (experimental). The MATLAB code for calculating the Γ factor is provided in Gerstner (2007). When Γ = 0, the prediction is no more than a chance, and when Γ = 1, the prediction is optimal. When dealing with several recordings of the same neuron, the Γ factor should be normalized with respect to the intrinsic reliability Γ_*in*_. The normalized mean Γ factor across *N*_*rep*_ repetitions of the same experiment is given as (Naud et al., 2009).


(7) $$\begin{array}{l} P_{A}=\frac{1}{N_{rep}}\overset{N_{rep}}{\underset{i=1}{\sum }}\frac{\Gamma_{i}}{\Gamma_{in}}, \end{array}$$

where Γ_*i*_ is the coincidence factor between the model spike train and the *i*-th recording. *N*_*rep*_ = 13 in QSNMC2009. The intrinsic reliability is calculated between the neuron recordings as the average Γ factor between each unique recording. It is formulated as (Naud et al., 2009).


(8) $$\begin{array}{l} \Gamma_{in}=\frac{2}{N_{rep}\left(N_{rep}-1\right)}\overset{N_{rep}}{\underset{i=1}{\sum }}\overset{N_{rep}}{\underset{j=i+1}{\sum }}\Gamma_{i,j}, \end{array}$$

where Γ_*i,j*_ is the coincident factor between recordings *i* and *j*.

## 4. Parameter Identification Search

### 4.1. Problem Formulation

**Figure 1:**
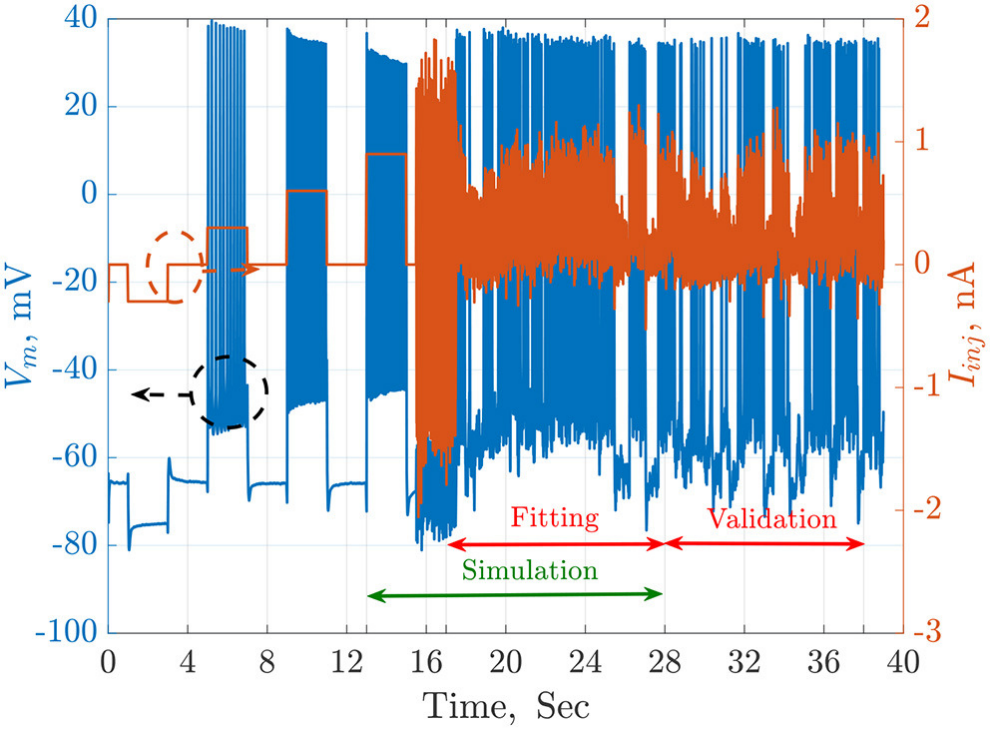
The injected current, *I*_*inj*_ in orange, and the recorded membrane potential, *V*_*m*_ in blue, of a single trial from the QSNMC2009 dataset. The dashed circles point each signal to its corresponding axis on the left or the right. The timeline is segmented into simulation, fitting, and validation periods. The simulation starts from an equilibrium point and not from the beginning to reduce the total time consumed by each agent during the search of the meta-heuristic optimization algorithms.

The available QSNMC2009 data includes an injected current and 13 membrane potential recordings stimulated by the same current. The time length of the online data is 38 s. In this work. the simulations are started at *t* = 13 s, which is an equilibrium point. The fitting duration is from *t* = 17.5 to *t* = 28 s, while the validation duration starts at *t* = 28 s to the end of the online data-set (*t* = 38 s). These time regions are illustrated in Figure 1.

Two optimization problems are considered in this work. Problem 1 is maximizing the average Γ factor across all recordings. This problem is formulated as


(9) $$\begin{array}{l} \underset{\text{x}}{\max}\frac{1}{N}\overset{N}{\underset{i=1}{\sum }}\Gamma_{i}, \end{array}$$

where **x** is the search vector, *N* is the number of recordings, which is 13 for QSNMC2009, and Γ_*i*_ is the calculated coincidence factor between the estimated spike times and the spike times of the *i*th recording.

The second problem, which is the one used in Rossant et al. (2010) and Lynch and Houghton (2015), is a set of independent optimization problems where each one is concerned with maximizing the coincidence factor between the estimated spike train and the spike train of one of the 13 recordings.


(10) $$\begin{array}{l} \underset{{\text{x}}_{i}}{\max}\Gamma_{i},1\leq i\leq N. \end{array}$$

where **x**_*i*_ is the search vector corresponding to recording *i*. The second problem formulation has *N* times the dimension of the first problem and the optimal parameters of the second problem are expected to differ between recordings.

MATLAB R2020a has been used to run the optimization procedures on IBEX HPC at KAUST. Each run was repeated 10 times in problem 2 to report the mean, standard deviation (std), coefficient of variation (CV=std/mean) for each parameter, and the fitting and validation coincidence factors, while each run was repeated 100 times for problem 1 to balance the total number of runs between both problems. A coincidence window of 4 ms was used throughout these optimizations to evaluate the coincidence factor (Γ). The simulation time step is 0.1 ms and the Euler numerical method is the one used to evaluate the estimated membrane potential. The GA algorithm is set to 100 generations as this is found to stall very early, while other algorithms are set to 10k iterations. The algorithms parameters are assumed as the default unless otherwise specified. The upper and lower limits for the parameters of each model are summarized in Table 1. The values for the upper and lower limits were inspired by the methodology of similar papers in the literature (Lynch and Houghton, 2015).

**Table 1:** The upper and lower bounds for the search space of the two models: aEIF and aTIF-W.

**aEIF**	**τ_*m*_, ms**	**τ_*w*_, ms**	**b**	***V*_*T*_, mV**	***V*_*r*_, mV**	***E*_*L*_, mV**	**α, mV**	**Δ_*T*_, mV**	***R*, MΩ**	**-**
Lb	1	80	0	−30	−100	−100	10	1	70	-
Ub	15	150	5	−10	−50	−50	40	5	200	-
**aTIF-W**	**τ_*m*_, ms**	**τ_*w*_, ms**	**τ_*t*_, ms**	**b**	**c**	***V*_*r*_, mV**	***E*_*L*_, mV**	**α, mV**	**β, mV**	**R, MΩ**
Lb	1	20	20	0	−3	−120	−120	0	0	70
Ub	15	150	150	5	3	−40	−40	40	40	200

### 4.2. Meta-Heuristic Search Algorithms

Meta-heuristic algorithms are categorized into four classes: the evolutionary algorithm-like genetic algorithm and differential evolution, the swarm intelligence algorithm-like particle swarm optimizer and artificial bee colony optimizer, the natural phenomena algorithm-like intelligent water drops and water cycle algorithm, and human-inspired algorithms such as the seeker optimization and soccer league competition (Abd Elaziz et al., 2021). In this work, we consider one algorithm of the first class (genetic algorithm), and the other four algorithms are of the second class.

#### 4.2.1. Genetic Algorithm

In the 1960s and 1970s, John Holland and his collaborators developed the genetic algorithm that models the biological evolution theory, and the concept of natural selection (Holland et al., 1992). The use of crossover, mutation, and selection was very new to artificial systems. Many updates were later made, and many variants were applied to a vast range of optimization problems in science and engineering. There are many advantages to using GA that includes dealing with complex problems, time-varying objective functions, discontinuous search space, and non-linear problems. A GA can also be parallelized easily, as its agents can evaluate the objective function independently, which is the case for most meta-heuristic optimization algorithms. On the other hand, there are disadvantages to a GA. For example, the many parameters of the algorithm need to be carefully tuned, such as the rate of mutation and selection criteria. A bad combination of these parameters and the optimization problem can make it difficult for the algorithm to converge to a reasonable solution (Yang, 2020). The algorithm has three main operations: selection, crossover, and mutation. In selection, the parents of the upcoming generation are selected from the current population. In the crossover, two parents are combined to produce the children of the next generation. The mutation is the process of randomly changing some features of the individual parents to produce children. In this work, we used the standard genetic algorithm in MATLAB.

#### 4.2.2. Cuckoo Search and Its Fractional Variant

The cuckoo search (CS) was innovated by Yang and Deb (2009) inspirited from the natural behavior of cuckoo breeding parasitism. In Gandomi et al. (2013), the authors modeled that behavior mathematically *via* three hypotheses: (I) each cuckoo lays one egg at a time, (II) a cuckoo puts an egg in a nest chosen randomly, and the fittest is retained for the next generation, and (III) the available host nests are bounded, and a host cuckoo can detect a stranger egg with a probability of *P*_*s*_ ∈ [0, 1] (*P*_*s*_ = 0.1 in this work). These behaviors can be formulated mathematically using *Levy* flights.

Later on, the fractional variant of the cuckoo search optimizer was introduced (Yousri and Mirjalili, 2020). The main idea was to fractionalize the exploration random walk in order to improve the diversification capabilities of the algorithm. With a fractional-order cuckoo optimizer, the memory effect is added to the classical CS to boost the random walk. The extra parameters given by fractional calculus are used to adaptively tune the harmonization between the global and local random walks. Based on Yousri and Mirjalili (2020), the recommended value of the derivative order is 0.3, with a memory length of four terms.

#### 4.2.3. Marine Predator Algorithm

The marine predators algorithm (MPA) is a biologically inspired optimization algorithm that has been recently proposed in Faramarzi et al. (2020). It simulates the interactions between marine preys and predators based on the governing policies for optimal foraging and memories in marine predators. For example, it has been found that marine predators use a *Levy* strategy and Brownian motion for areas with low and high concentrations of prey, respectively. However, they use both random walk strategies during their lifetime while traversing different habitats. Predators use their good memory to their advantage by reminding themselves and their associates of the locations where their hunt ended successfully in the past.

As in most meta-heuristic algorithms, the MPA population is initialized by drawing from a uniform distribution within the feasible region of the search space. After that, two important matrices are constructed: the Prey matrix and the Elite matrix. Both matrices have the dimension *n*×*d*, where *n* is the number of search agents, and *d* is the dimension of the search space. Initially, the rows of the Prey matrix contain the positions of the prey from the initialization step. After that, the Prey matrix update strategy will be different according to the phase of the optimization. At each iteration, the Elite matrix is constructed by replicating the position of the top predator across all rows.

The optimization process of MPA is equally divided into three phases based on the velocity ratio between predators and prey. In Phase 1, when the predator is moving faster than the prey, in the exploration phase, the prey moves in Brownian motion during the first third of the iterations. During the second phase, a transition from exploration to exploitation occurs. Half of the population is in exploration modes, and the other half is in exploitation mode. During the last phase, the predators switch to *Levy* flight instead of Brownian motion in order to emphasize the exploitation.

#### 4.2.4. Particle Swarm Optimization

Kennedy and Eberhart developed particle swarm optimization in 1995 (Kennedy and Eberhart, 1995). Since then, it became one of the most widely used swarm intelligence algorithms, as it is straightforward and flexible. The algorithm uses randomness and communication between its particles to update their location in the search space. It is easy to implement due to the lack of encoding and decoding of parameters when compared to a GA. The idea behind PSO has inspired many new algorithms, and this can be seen in the similarity between the structure of these new algorithms and PSO (Yang, 2020). The algorithm is summarized as follows: the particles move in the search space in steps. At each of these steps, the objective function is evaluated, and the new location of each particle is calculated based on this evaluation. The movement is towardz a combination of the best location that the particle has had so far and the global best location of all particles. In this work, we used the original MATLAB implementation of the PSO algorithm.

## 5. Results and Discussion

In this section, we discuss the comparative results of the aforementioned optimization algorithms on the QSNMC2009 dataset. Besides, in order to facilitate the reproducibility of the results, the codes are available online on the project website^1^.

Table 2 summarizes the fitting results of Optimization problem 1 using the aEIF model. The most variable parameter across iterations is *b* for all algorithms. On the other hand, the least variable parameter is *R* for all algorithms except the MPA, whose least variable parameter is τ_*m*_. The MPA, CS, and FOCS achieved a higher fitting coincidence factor than GA and PSO. The best validation coincidence factor is achieved by the MPA, followed closely in second place by FOCS. The variability in the fitting coincidence factor is less than the variability of the validation coincidence factor.

**Table 2:** Summary of fitted parameters; the fitting and validation Γ factor of the aEIF model in problem 1.

		**τ_*m*_**	**τ_*w*_**	**b**	***V*_*T*_**	***V*_*r*_**	***E*_*L*_**	**α**	**Δ_*T*_**	**R**	**Γ fitting**	**Γ validation**
GA	Mean	8.375	101.656	1.358	−18.566	−82.589	−63.669	20.832	1.794	174.901	0.475	0.358
	CV	23.42E-02	14.59E-02	59.59E-02	−29.93E-02	−13.87E-02	−16.58E-02	27.11E-02	36.17E-02	10.58E-02	01.67E-02	06.02E-02
PSO	Mean	8.430	103.307	1.051	−20.457	−79.336	−72.426	18.938	1.652	179.693	0.483	0.359
	CV	23.45E-02	18.14E-02	84.87E-02	−31.42E-02	−20.64E-02	−20.58E-02	33.54E-02	45.74E-02	12.64E-02	02.23E-02	06.98E-02
MPA	Mean	9.562	93.319	0.552	−22.811	−70.357	−72.633	20.805	1.139	170.759	**0.513**	**0.399**
	CV	09.10E-02	14.99E-02	65.13E-02	−26.57E-02	−18.63E-02	−13.23E-02	18.07E-02	23.51E-02	12.99E-02	01.15E-02	06.87E-02
CS	Mean	9.683	110.846	0.348	-19.979	−77.546	−72.761	18.227	1.380	170.425	0.510	0.382
	CV	20.45E-02	21.26E-02	52.49E-02	−26.42E-02	−19.68E-02	−16.46E-02	32.14E-02	35.58E-02	14.45E-02	01.19E-02	08.48E-02
FOCS	Mean	9.195	94.328	0.575	−19.520	−69.542	−68.372	19.953	1.285	165.677	0.507	0.392
	CV	14.42E-02	16.49E-02	67.36E-02	−29.77E-02	−21.27E-02	−12.97E-02	21.60E-02	28.37E-02	13.34E-02	01.07E-02	07.89E-02

*The best fitting and validation coincidence factors are written in bold*.

Table 3 summarizes the results of problem 2 using the aEIF model. The most and least variable parameters across all algorithms are *b* and *R*, respectively, except for FOCS, whose least variable parameters are *R* and τ_*w*_. The MPA and CS achieved the best fitting coincidence factor, followed closely by the FOCS, while the PSO and GA achieved the lowest values for the fitting coincidence factor. The validation coincidence factor is approximately the same for all algorithms. The variability of the fitting coincidence factor is very close between algorithms, while the variability of the validation coincidence factor is also the same, except that for the GA, which is lower than the rest of the algorithms.

**Table 3:** Summary of fitted parameters; the fitting and validation Γ factor of the aEIF model in problem 2.

		**τ_*m*_**	**τ_*w*_**	**b**	***V*_*T*_**	***V*_*r*_**	***E*_*L*_**	**α**	**Δ_*T*_**	**R**	**Γ fitting**	**Γ validation**
GA	Mean	8.1890	103.9098	1.3953	−19.4152	−80.0093	−65.8482	19.7755	2.0600	174.5987	0.5112	**0.3597**
	CV	21.38E-02	16.22E-02	71.71E-02	-29.49E-02	−17.70E-02	−17.82E-02	30.00E-02	40.96E-02	10.20E-02	06.09E-02	09.62E-02
PSO	Mean	8.7731	104.9213	1.2591	−19.7134	−77.1091	−70.4785	18.9612	1.7642	180.2081	0.5319	0.3505
	CV	27.89E-02	19.08E-02	88.48E-02	−31.61E-02	−20.37E-02	−22.51E-02	37.61E-02	55.09E-02	11.80E-02	05.91E-02	11.75E-02
MPA	Mean	9.5678	98.4903	0.9829	−19.2104	−77.8583	−68.8243	18.2399	1.2991	168.0283	0.5752	0.3531
	CV	25.79E-02	20.06E-02	01.03E+00	−27.69E-02	−21.68E-02	−20.05E-02	36.77E-02	26.69E-02	15.00E-02	05.03E-02	13.18E-02
CS	Mean	10.3605	110.9655	0.5567	−19.7346	-80.3343	-72.0564	16.3476	1.5744	167.9558	**0.5761**	0.3464
	CV	26.23E-02	20.36E-02	01.42E+00	-28.67E-02	-17.98E-02	−21.46E-02	29.61E-02	38.67E-02	15.45E-02	05.14E-02	11.97E-02
FOCS	Mean	8.8954	97.7924	0.9237	−18.7916	−75.4606	−68.0144	18.2478	1.3543	164.4323	0.5656	0.3576
	CV	25.46E-02	17.02E-02	01.02E+00	−30.82E-02	−21.97E-02	−18.85E-02	36.52E-02	33.21E-02	17.67E-02	06.57E-02	11.55E-02

*The best fitting and validation coincidence factors are written in bold*.

Table 4 summarizes the optimal parameters of the aTIF-W model when used in problem 1. The best fitting coincidence factor is achieved by the MPA followed by CS and FOCS. The best validation coincidence factor is achieved by the GA, followed by the PSO. The most variable parameter for all algorithms is β, while the least variable parameter is *R*.

**Table 4:** Summary of fitted parameters; the fitting and validation Γ factor of the aTIF-W model in problem 1.

		**τ_*m*_**	**τ_*w*_**	**τ_*t*_**	**b**	**c**	***V*_*r*_**	***E*_*L*_**	**α**	**β**	**R**	**Γ fitting**	**Γ validation**
GA	Mean	7.9430	70.6210	79.1497	2.7231	−1.2536	−77.6165	−60.9129	13.9722	8.7192	166.9074	0.4675	**0.3412**
	CV	25.85E-02	38.73E-02	52.12E-02	41.66E-02	−65.35E-02	−27.00E-02	−23.99E-02	60.88E-02	85.39E-02	14.40E-02	02.07E-02	09.00E-02
PSO	Mean	7.4221	86.4086	81.4162	3.4869	−1.7659	−81.6557	−67.4959	13.1968	11.4470	175.2411	0.4749	0.3396
	CV	29.18E-02	33.55E-02	57.15E-02	36.47E-02	−51.76E-02	−29.70E-02	−27.67E-02	78.46E-02	01.04E+00	15.90E-02	02.09E-02	08.62E-02
MPA	Mean	7.5485	41.4732	66.6549	1.9290	−0.7909	−69.2158	−58.6748	26.2633	3.1894	154.1694	**0.5075**	0.3380
	CV	29.14E-02	40.34E-02	68.07E-02	52.24E-02	−96.37E-02	−39.93E-02	−20.33E-02	29.76E-02	01.08E+00	18.71E-02	01.51E-02	08.50E-02
CS	Mean	9.1429	57.5165	104.7507	2.4674	-1.5740	−85.3730	−63.9033	21.5919	5.3737	161.8457	0.5013	0.3292
	CV	30.75E-02	47.19E-02	42.51E-02	44.72E-02	−60.60E-02	−33.66E-02	−31.83E-02	50.22E-02	01.15E+00	20.23E-02	02.09E-02	11.51E-02
FOCS	Mean	8.0000	48.9272	80.9417	1.9770	−1.1475	−73.4521	−57.6855	18.5179	5.8169	141.8364	0.5023	0.3305
	CV	34.61E-02	44.12E-02	63.21E-02	41.30E-02	−69.68E-02	−41.00E-02	−24.72E-02	45.58E-02	98.75E-02	19.39E-02	01.43E-02	10.29E-02

*The best fitting and validation coincidence factors are written in bold*.

Table 5 summarizes the results of problem 2 when solved using the aTIF-w model. The best fitting coincidence factor is obtained by the MPA, and the best validation coincidence factor is obtained by the GA. The variability in the fitting coincidence factor result is the least for FOCS, followed by CS and MPA in ascending order. The most and least variable parameters are β and *R*, respectively, for all algorithms.

**Table 5:** Summary of fitted parameters; the fitting and validation Γ factor of the aTIF-W model in problem 2.

		**τ_*m*_**	**τ_*w*_**	**τ_*t*_**	**b**	**c**	***V*_*r*_**	***E*_*L*_**	**α**	**β**	**R**	**Γ fitting**	**Γ validation**
GA	Mean	8.068	74.428	75.307	2.630	−1.412	−85.300	−64.212	14.906	11.479	170.415	0.503	0.341
	CV	29.44E-02	36.98E-02	51.35E-02	50.72E-02	−65.92E-02	−27.07E-02	−27.12E-02	63.38E-02	80.73E-02	13.74E-02	06.09E-02	11.72E-02
PSO	Mean	8.253	87.115	84.494	3.066	−1.787	−81.297	−63.080	15.359	10.900	169.019	0.524	**0.343**
	CV	30.48E-02	34.27E-02	53.99E-02	44.59E-02	−53.32E-02	−30.43E-02	−29.14E-02	61.91E-02	99.57E-02	16.49E-02	05.88E-02	13.04E-02
MPA	Mean	9.026	70.943	92.815	1.986	−1.161	−85.569	−60.253	18.932	10.605	160.667	**0.575**	0.338
	CV	34.04E-02	39.42E-02	53.08E-02	51.45E-02	−65.48E-02	−33.26E-02	−29.93E-02	59.05E-02	01.00E+00	17.81E-02	04.90E-02	16.46E-02
CS	Mean	10.108	74.807	115.447	2.027	−1.770	−87.567	−67.829	19.181	7.949	160.696	0.570	0.327
	CV	27.35E-02	46.02E-02	31.51E-02	58.56E-02	−52.08E-02	−29.70E-02	−34.09E-02	62.07E-02	01.21E+00	20.70E-02	04.75E-02	22.95E-02
FOCS	Mean	8.839	72.563	93.087	2.080	−1.467	−83.416	−61.686	16.100	10.961	153.339	0.565	0.337
	CV	32.67E-02	37.30E-02	48.82E-02	54.48E-02	−61.42E-02	−28.70E-02	−32.32E-02	63.19E-02	94.62E-02	18.27E-02	04.68E-02	19.18E-02

*The best fitting and validation coincidence factors are written in bold*.

**Figure 2:**
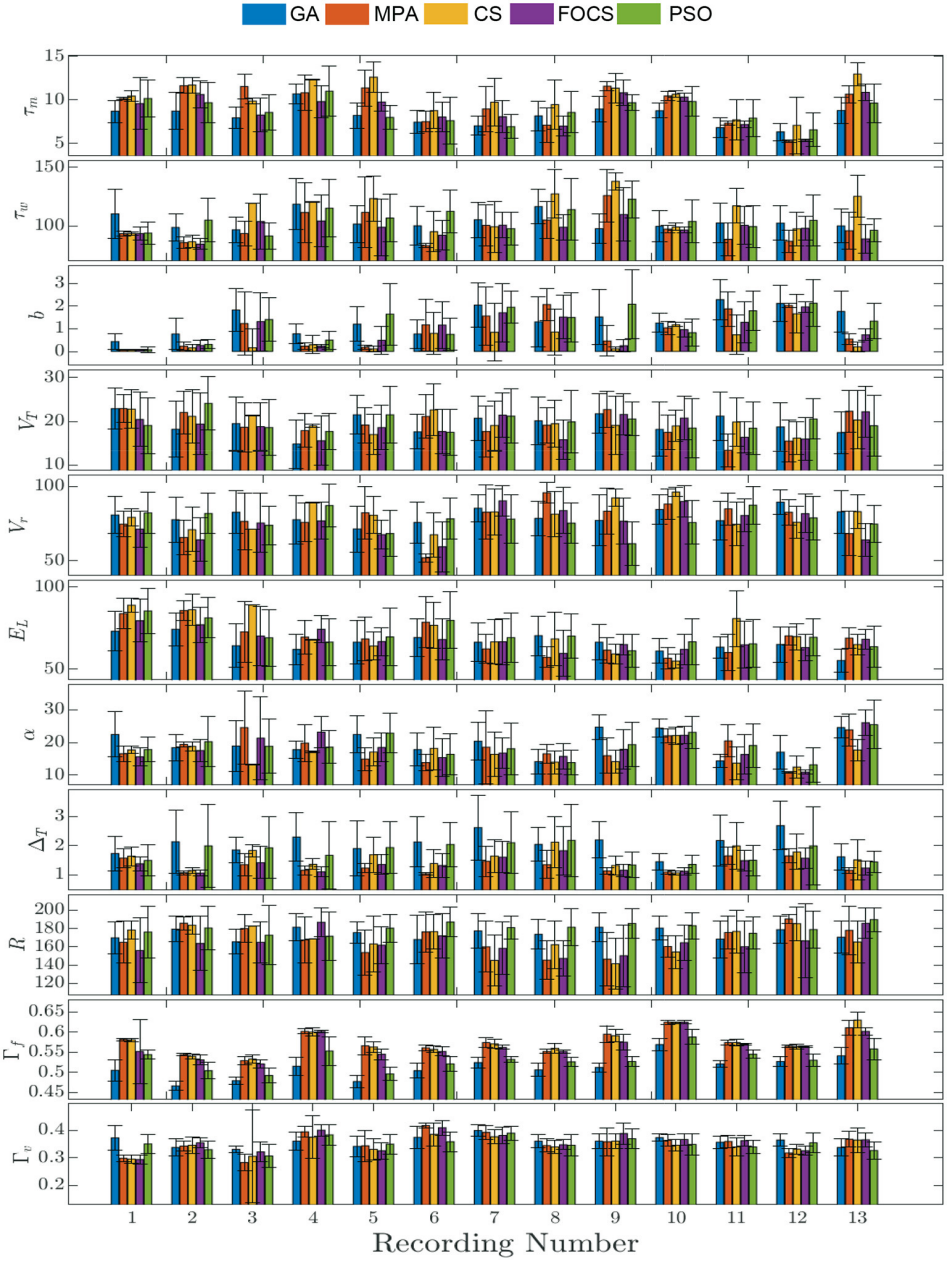
Parameter mean and standard deviation across 10 independent runs for the aEIF model using 5 optimization algorithms for each recording. Each column depicts the mean value of the parameter and the standard deviation is shown as a vertical line segment whose center is at the mean.

Figure 2 summarizes the results of problem 2 and provides more details into the behavior of each record individually. The solid bars are the mean value of the parameter, while the vertical line segments are the error bounds based on the standard deviation. The highest fitting coincidence factor is achieved with Record 10, where the MPA, CS, and FOCS are more consistent than the PSO and GA. On the other hand, the highest validation coincidence factor is achieved with Record 6 in the case of the MPA and FOCS algorithms, and the MPA is more consistent. The parameter *b* has a larger value in the case of record 12 when compared to other records. The lowest membrane time constant, τ_*m*_, value is seen with Record 12, but the same record shows the highest membrane resistor, *R*, value.

**Figure 3:**
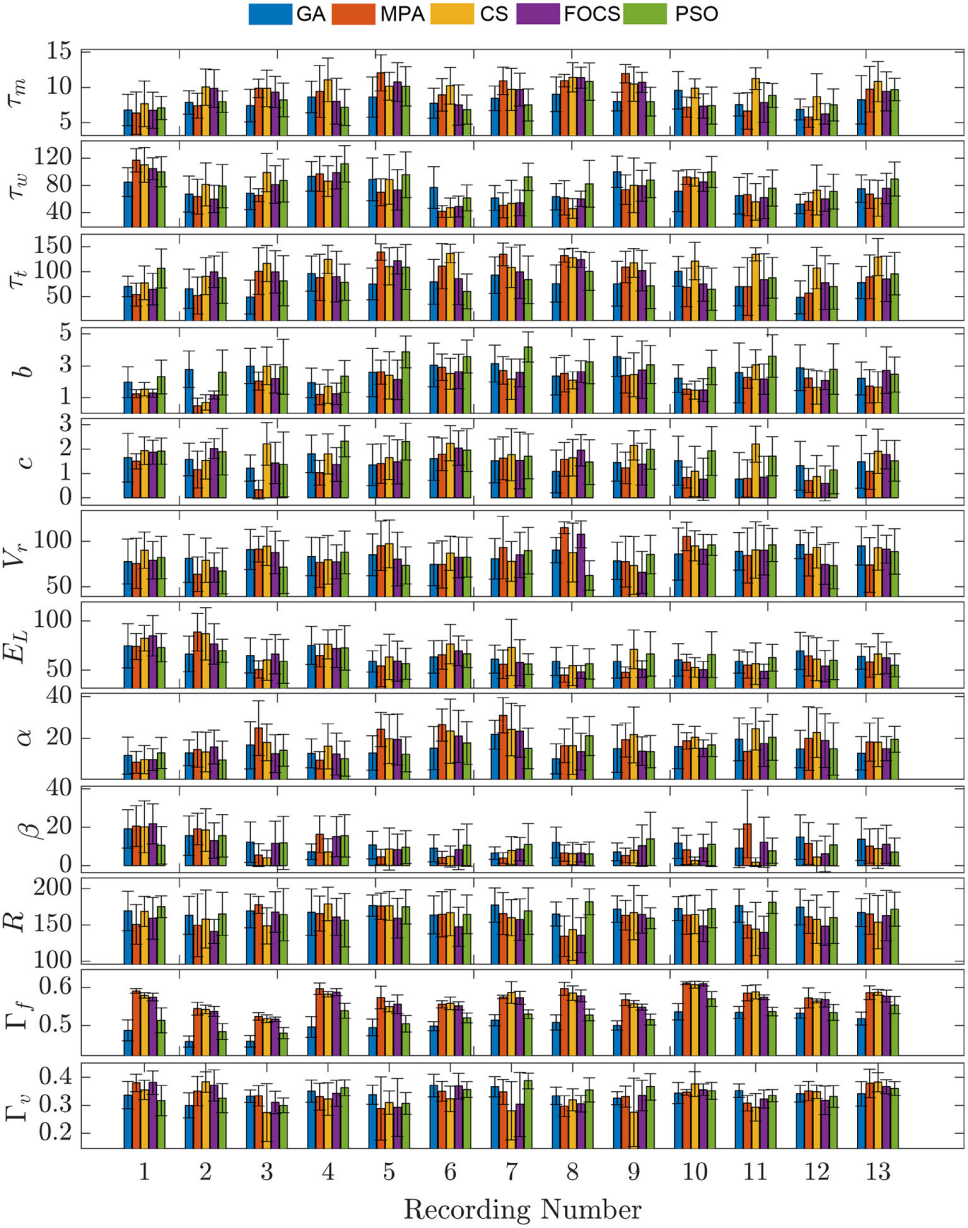
Parameter mean and standard deviation across 10 independent runs for the aTIF-W model using 5 optimization algorithm for each recording.

In Figure 3, problem 2 results when using the aTIF-W model are summarized for each record. Record 10 shows the highest fitting coincidence factor values with remarkable consistency from the MPA, CS, and FOCS, while Record 13 achieves higher values for the validation coincidence factor. Parameter β has lower values than parameter α for all records except the first two.

**Figure 4:**
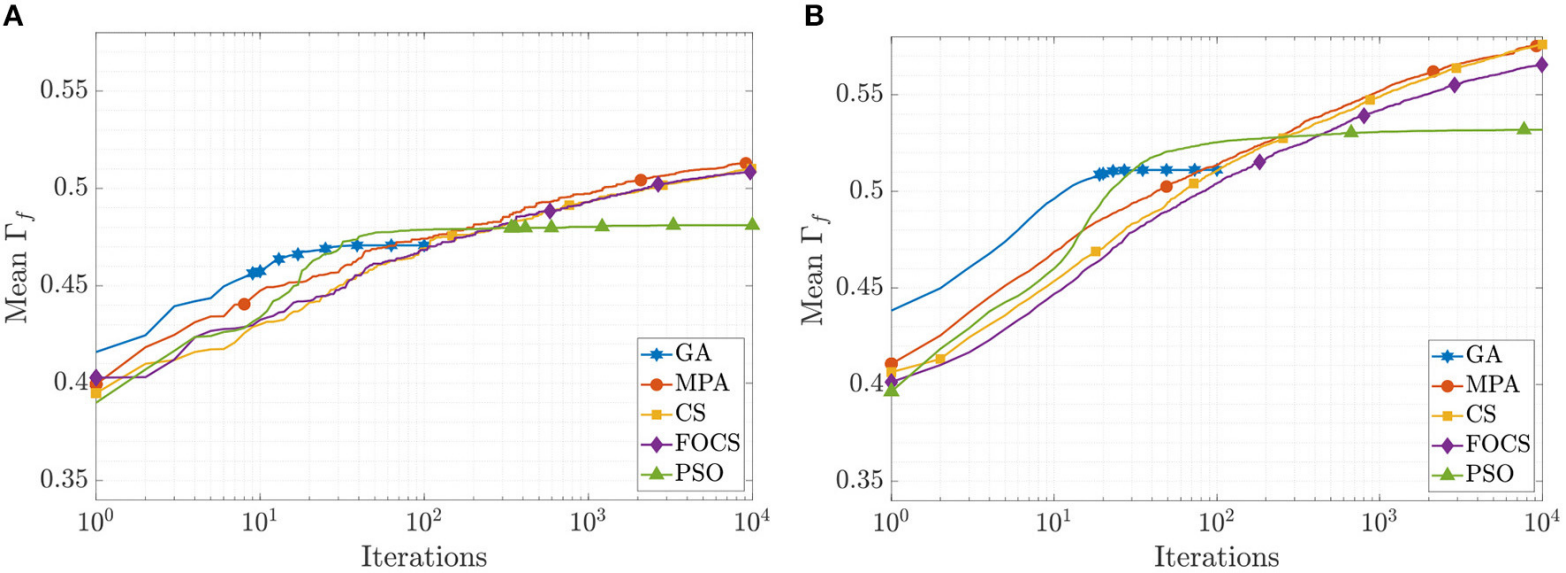
CG curves of the aEIF model fitting results using the QSNMC2009 dataset. **(A)** Problem 1: maximizing mean Γ factor. **(B)** Problem 2: maximizing Γ factor for each record individually.

The mean convergence curves for problems 1 and 2 using the aEIF model are illustrated in Figure 4. In the case of problem 1, at iteration 50, the GA reached its optimal value, and before iteration 1, 000, the PSO reached Γ_*f*_ = 0.482, which is 99.8% of its final value. The CS and FOCS curves moved side by side, but at the end, the CS achieved a slightly higher coincidence factor than the FOCS. At iteration 2, 000, the MPA, CS, and FOCS are at ≈98% of their final values. For problem 2, the behavior of the GA and PSO is the same as in problem 1. The MPA and CS convergence curves are very close. At iteration 2, 000, the MPA, CS, and FOCS are at ≈ 97% of their final values.

**Figure 5:**
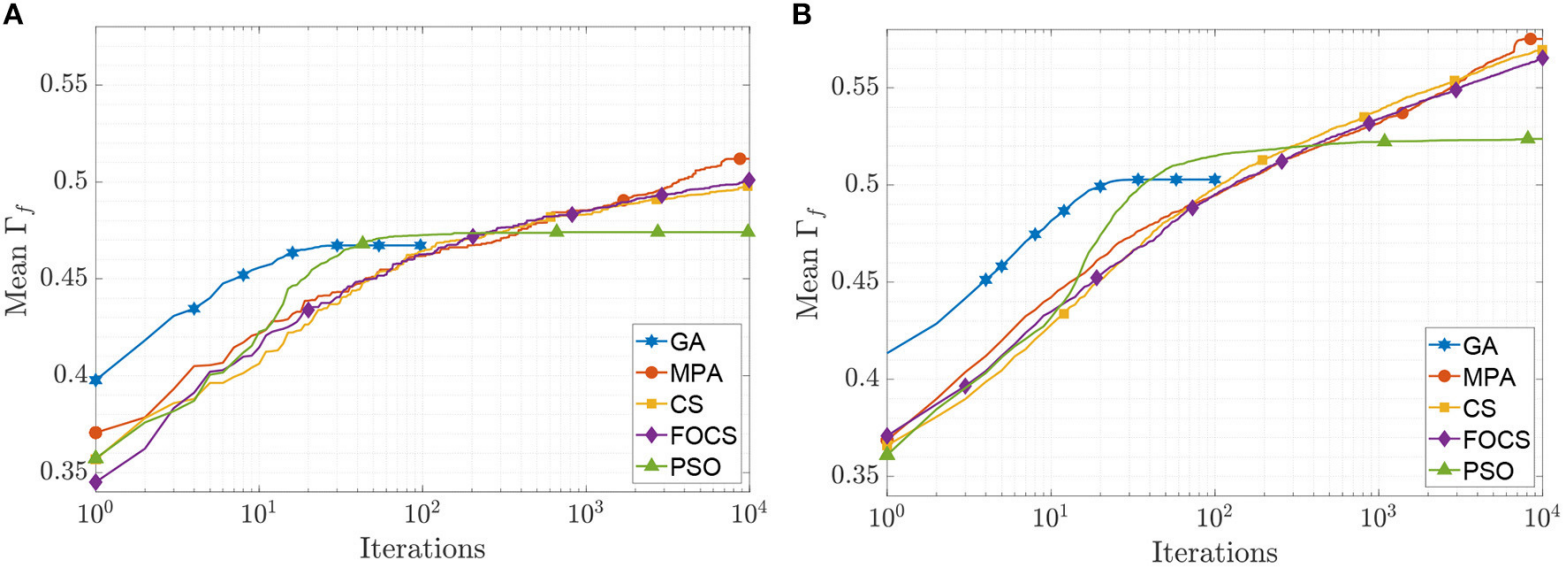
CG curves of the aTIF-W model fitting results using the QSNMC2009 dataset. **(A)** Problem 1: maximizing mean Γ factor. **(B)** Problem 2: maximizing Γ factor for each record individually.

The mean convergence curves of the aTIF-W model when used to solve problems 1 and 2 are depicted in Figure 5. The GA achieves its final value within 40 iterations, while the PSO achieves its 99.7% of its final value at iteration 500. At iteration 2, 000, the MPA achieves 96.7% of its final value, and the CS and FOCS arrive at 97.6% of their final values. For problem 2, the GA finishes at 34 iterations, while the PSO achieves 99.5% of its final value at iteration number 1, 000. At iteration 2, 000, the MPA, CS, and FOCS arrive at ≈95% of their final values.

In summary, the fitting coincidence factor is always greater than the validation coincidence factor. The fitting coincidence factor for problem 1 is lower than that of problem 2 for the same model, regardless of the optimization algorithm used. A higher fitting coincidence factor does not necessarily imply a higher validation coincidence factor for this particular dataset. The aEIF model has a higher fitting and validation coincidence factor in problem 1 than the aTIF-W model, although the fitting coincidence factor of the MPA is higher in aTIF-W, and FOCS has a nearly equal fitting coincidence factor in both models. For problem 2, the validation coincidence factor is always lower in the case of aTIF-W when compared to aEIF. However, the fitting coincidence factor is the same for the MPA, CS, and FOCS across both models, while the GA and PSO achieve a higher fitting coincidence factor in the case of aEIF. Parameter variation is higher in the aTIF-W model than in the aEIF model. The GA and PSO converge faster than the MPA, CS, and FOCS, but at lower objective function values. Consistency is clearly seen in the fitting coincidence factor results; however, parameter results are less consistent even in the case of the new meta-heuristic algorithms: the MPA, CS, and FOCS. Also, the small variability of the *R* parameter may suggest detrending it, removing it from the search space, which may result in more consistent fitting results (Pozzorini et al., 2013).

Table 6 summarizes the distribution of the optimal fitting and validation coincidence factors calculated for each record. The mean fitting coincidence factor is higher in problem 1 when compared to problem 2 for all algorithms and models. This is opposed to what is reported in Tables 2–5, where the reported values for the coincidence factor for problem 2 are for the record that the optimizer used only, not for the 13 recordings. Furthermore, the variability of the fitting coincidence factor is lower for problem 1 than for problem 2. The mean validation coincidence factor is higher in problem 1 than in problem 2 in the case of the aEIF model, while they are between 0.34 and 0.32 in the case of the aTIF-W model. The fitting coincidence factor rarely exceeds 0.6 for any record, while the validation coincidence factor rarely exceeds 0.5 for any record. The largest difference between fitting and validation coincidence factors is 0.172 in the CS and FOCS for the aTIF-W model in problem 1, which is the highest case for the other algorithms too, and the smallest difference is 0.09, which is seen for the GA in the aEIF model in problem 1.

**Table 6:**
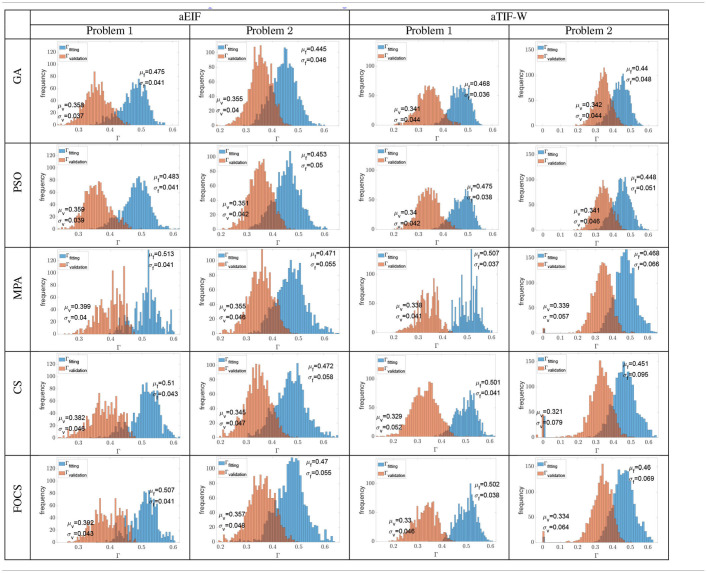
Histograms of all the fitting and validation coincidence factors achieved from all the independent trials when calculated for each experimental recording (cross validation).

## 6. Conclusion

Three algorithms were introduced to the problem of fitting spike trains of experimental neuron data. These algorithms are the MPA, CS, and FOCS. The issue was formulated into two optimization problems. The coincidence window achieved by the second problem was higher than the first problem due to the increased number of parameters, and the aEIF model obtained better fitness function values than the aTIF-W model. However, upon performing cross-validation on all 13 recordings of the experimental dataset, the fitting coincidence factors for problem 2 decreased below those of problem 1. The newly utilized algorithms showed consistent fitness function results across independent trials. However, parameter consistency across independent trials was not achieved. This explains why many papers in the literature only report the fitness values, not the parameter values. Moreover, the objective function values were at least at 95% of their final values at 2, 000 iterations. This means that the number of iterations used in this work can be reduced significantly without sacrificing the optimal objective function value.

The common problem formulation used in the literature is problem 2, which failed to achieve a higher fitting coincidence factor upon cross-validation. This is despite the larger search space of problem 2 when compared to problem 1. Based on this observation, researchers should consider the formulation mentioned in problem 1 and optimize the mean coincidence factor instead of optimizing the coincidence factor of each record individually, as done in problem 2. Another important note is that GA achieved the highest validation coincidence factor in case of aEIF model on problem 2 and the aTIF-W model on problem 1 despite using smaller number of iterations than other algorithm in this study. Also, the only case when an algorithm achieved the highest fitting and validation coincidence factor is for the aEIF model in problem 1, and it was achieved by the MPA. In the other cases, the best validation and fitting performance were not achieved by the same algorithm, which is a sign of overfitting. The improvement that is achieved by the new algorithms in the fitting coincidence factor was less than 10% when compared to the more common algorithms for this problem (the GA and PSO). However, the aim of this work is to introduce a modified methodology other than the ones in the literature, which mainly depend on GA and PSO algorithms, which is a similar motivation for the work in Marín et al. (2021). Thus, this opens the door for researchers to investigate other newly introduced meta-heuristic algorithms to tackle the two problems presented in this paper. Algorithms with faster convergence and a lower number of function evaluations are recommended.

## Data Availability Statement

Publicly available datasets were analyzed in this study. This data can be found at: https://github.com/INCF/QSNMC.

## Author Contributions

AA and MF conceptualized the work. AA worked on the experiments and simulations and wrote of the first draft. AA, MF, and AE analyzed and discussed the results. MF and AE revised the manuscript. AE supervised the research. All authors contributed to the article and approved the submitted version.

## Funding

The authors are thankful to King Abdullah University of Science and Technology (KAUST), Kingdom of Saudi Arabia, for funding this work.

## Conflict of Interest

The authors declare that the research was conducted in the absence of any commercial or financial relationships that could be construed as a potential conflict of interest.

## Publisher's Note

All claims expressed in this article are solely those of the authors and do not necessarily represent those of their affiliated organizations, or those of the publisher, the editors and the reviewers. Any product that may be evaluated in this article, or claim that may be made by its manufacturer, is not guaranteed or endorsed by the publisher.

## Footnotes

^1^ https://mefouda.me/project/fractional-order-spiking-systems/

## Supplementary Material

Supplementary Data Sheet 1 Excel workbook of the aEIF model's problem 1 results.

Supplementary Data Sheet 2 Excel workbook of the aEIF model's problem 2 results.

Supplementary Data Sheet 3 Excel workbook of the aTIF-W model's problem 1 results.

Supplementary Data Sheet 4 Excel workbook of the aTIF-W model's problem 2 results.
